# Thermo-pH dual-responsive nanocarriers enable multiscale-controlled resveratrol release via molecular remodeling and electronic decoupling

**DOI:** 10.1016/j.isci.2026.115440

**Published:** 2026-03-23

**Authors:** Qijiang Shu, Zedong Lin, Wenjuan Zhao, Xiaokun Hua, Pengru Huang, Yong Qiu, Li Li, Yunpeng Luan

**Affiliations:** 1 Institute of Information, Yunnan University of Chinese Medicine, Kunming 650500, China; 2 Yunnan Engineering Research Centre of Preventive Treatment of Chinese Medicine, Kunming 650500, China; 3 Yunnan Provincial Hospital of Traditional Chinese Medicine, Yunnan University of Chinese Medicine, Kunming 650500, China; 4 Kunming Municipal Hospital of Traditional Chinese Medicine, Yunnan University of Chinese Medicine, Kunming 650500, China; 5 Guangdong Provincial Key Lab of Nano-Micro Materials Research, School of Chemical Biology and Biotechnology, Shenzhen Graduate School, Peking University, Shenzhen 518055, China; 6 Department of Science and Technology, Yunnan University of Chinese Medicine, Kunming 650500, China; 7 Guangxi Key Laboratory of Information Materials and Guangxi Collaborative Innovation Center of Structure and Property for New Energy and Materials, School of Material Science & Engineering, Guilin University of Electronic Technology, Guilin 541004, China

**Keywords:** drug delivery system, molecular modeling, nanomaterials

## Abstract

Resveratrol (RES) suffers from poor solubility and non-specific distribution, motivating the development of delivery systems with controllable stability and stimulus responsiveness. Here, we construct a hybrid nanocarrier composed of poly(N-isopropylacrylamide) and poly(L-lysine) that exhibits coupled temperature and pH responsiveness. Multiscale molecular simulations in explicit solvent, combined with quantum chemical optimization and free energy analysis, elucidate how external stimuli regulate carrier structure and RES release. At ambient conditions, RES is stably encapsulated within compact polymer clusters through cooperative noncovalent interactions. Elevated temperature induces hydrophobic collapse and structural reorganization, weakening polymer-drug interactions and enabling thermo-responsive release. Under acidic conditions, protonation of lysine segments triggers electrostatic repulsion and cluster dissociation, promoting drug desorption. These coordinated structural and energetic changes define a stimulus-driven “recognition-reconstruction-release” mechanism with tunable and predictable release behavior. Our findings provide molecular-level design principles for programmable nanocarriers targeting polyphenolic therapeutics.

## Highlights

• Thermo and pH dual responsive nanocarrier enables controlled RES release
• Multiscale simulations reveal thermodynamically stabilized encapsulation
• Hydrophobic collapse and protonation induced electrostatics trigger release
• Establishes molecular design principles for programmable polyphenol delivery

## Graphical abstract

**Figure:**
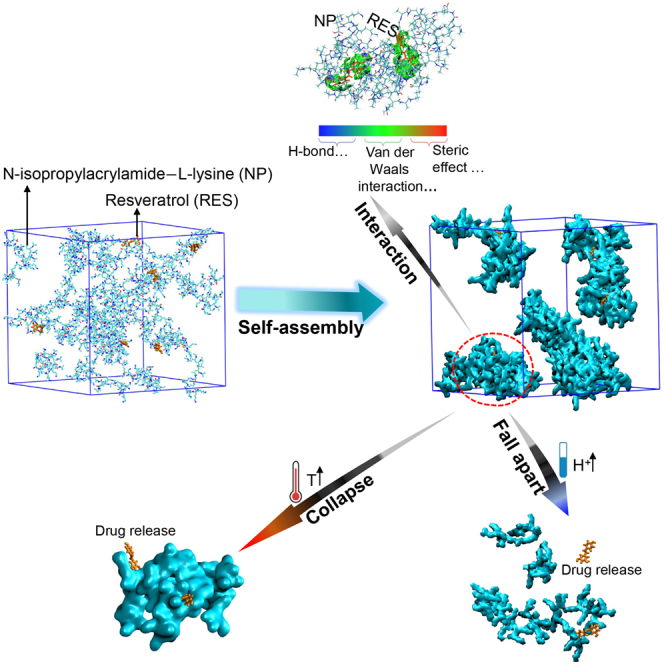

## Introduction

The high prevalence of major diseases such as chronic inflammation, metabolic disorders, and tumors continues to pose a severe threat to global human health, underscoring an urgent need for therapeutic strategies that are both effective and safe.^1^^,^^2^^,^^3^ In this context, naturally derived polyphenolic compounds have attracted considerable attention in drug development due to their broad pharmacological activities, low toxicity, and abundant natural availability. Resveratrol (RES), a plant-derived stilbene natural product, is regarded as a highly promising therapeutic candidate owing to its notable anti-inflammatory, antioxidant, cardiovascular protective, and antitumor properties.^4^^,^^5^^,^^6^

Despite exhibiting outstanding pharmacological effects *in vitro* and in animal models, the clinical translation of RES is severely hindered by multiple unfavorable physicochemical and pharmacokinetic properties.^7^ Its extremely low aqueous solubility, limited bioavailability, poor plasma stability, and rapid metabolism impede effective accumulation and controlled release *in vivo*, significantly restricting its therapeutic potential.^8^^,^^9^ To overcome these challenges, various drug delivery systems (DDS) have been explored, including liposomes,^10^ polymeric micelles,^11^ metal-organic frameworks (MOFs), and functionalized nanoparticles.^12^^,^^13^ Recent studies indicate that precise structural modulation of polymeric nanocarriers significantly improves the delivery performance of RES without altering its intrinsic chemical structure. For instance, nanosponge systems constructed via β-cyclodextrin crosslinking markedly enhance the aqueous solubility and dispersion stability of RES. By tuning the crosslinking density, these systems enable effective regulation of release kinetics and exhibit predictable sustained-release behavior under simulated physiological conditions.^14^ Further investigations systematically compare the influence of structural parameters of various nanocarriers on delivery performance and demonstrate that structural rearrangement and relaxation processes in aqueous environments closely correlate with the release rate of RES. These studies also validate, in cellular and animal models, the enhancement of drug bioavailability and therapeutic efficacy achieved through optimized carrier design.^15^ Despite these significant advances in material design and delivery optimization, current analyses remain largely based on macroscopic release profiles and experimental characterization data. A systematic and in-depth mechanistic elucidation of how nanocarrier structural evolution regulates the molecular-level processes of recognition, encapsulation, and detachment of RES remains lacking. This critical knowledge gap, to a considerable extent, limits the rational design and precise regulation of highly efficient and intelligent DDS architectures.

To address the difficulty of precisely regulating drug release behavior within complex pathological microenvironments, stimulus-responsive intelligent DDS emerge in recent years as a major research focus in precision medicine, owing to their ability to sense subtle environmental variations *in vivo* and trigger controllable release.^16^^,^^17^^,^^18^ Among these, thermoresponsive polymers-particularly poly N-isopropylacrylamide (NIPAM) with a lower critical solution temperature (LCST) near human body temperature-have attracted widespread attention in thermo-sensitive DDS due to their hydrophobic phase transition triggered by slight physiological temperature elevations, enabling controlled drug release.^19^^,^^20^^,^^21^ Concurrently, pH-responsive materials exploit ionization under acidic conditions to specifically recognize the acidic microenvironment characteristic of cancerous or inflamed tissues, thereby enhancing DDS targeting efficiency.^22^^,^^23^ Various stimulus-responsive strategies are introduced to construct multi-responsive DDS.^24^ However, existing studies generally confront a fundamental challenge of insufficient synergistic regulation: distinct responsive units are often combined through simple functional superposition without molecular-scale coupling design. This limitation frequently results in mutual interference between response behaviors, unpredictable release kinetics, and even potential risks to structural stability and biosafety.^25^^,^^26^^,^^27^ Accordingly, achieving synergistic regulation of multiple responsive behaviors within a unified molecular framework—while maintaining biocompatibility and structural stability—becomes a central scientific issue in the rational design of multi-responsive DDS architectures.

To achieve enhanced multi-responsiveness and improved system stability, it is imperative to incorporate structural units that combine robust biocompatibility with precise stimulus sensitivity. Poly-L-lysine (PLL), a cationic polypeptide derived from natural sources, emerges as a highly promising pH-responsive component.^28^^,^^29^ As a naturally derived cationic polypeptide polymer, PLL undergoes protonation of its side-chain amino groups under mildly acidic conditions, thereby conferring explicit and tunable pH-responsive characteristics to the material. This ionization process not only facilitates modulation of drug release kinetics but also strengthens electrostatic interactions with drug molecules or carrier frameworks, enhancing overall system stability and controllability. In addition, PLL is biodegradable and well-tolerated by biological tissues, and its degradation products-lysine monomers-can be directly metabolized by the body, substantially reducing the long-term risk of systemic toxicity.^30^ Moreover, the PLL backbone is densely populated with primary amine groups, providing abundant reactive sites for subsequent functionalization and the construction of targeting ligands. This structural feature endows the system with excellent scalability and engineering versatility, facilitating the development of customizable and application-specific drug delivery platforms.^31^

Existing studies indicate that NIPAM, as a thermoresponsive polymer, has been applied to a certain extent in RES delivery and exhibits relatively mature performance in temperature-triggered behavior and controlled release. However, these investigations predominantly focus on experimental construction and macroscopic evaluation of release profiles, while a systematic elucidation of molecular conformational evolution and drug-carrier interaction mechanisms during the temperature-triggering process remains lacking.^32^ In contrast, although PLL demonstrates pronounced advantages in pH responsiveness, biosafety, and structural tunability, its application in RES delivery systems remains highly limited.^33^ Against this background, the functionally complementary integration of NIPAM and PLL holds promise for achieving synergistic regulation of temperature- and pH-responsive behaviors within RES delivery platforms. Such an approach represents a rational pathway for exploring, at the molecular level, the structure-property-release relationships of multi-stimuli-responsive DDS specifically adapted to RES.

Overall, this study builds upon a systematic analysis of the requirements for efficient RES delivery and focuses on the synergistic regulation of thermos-responsiveness, pH responsiveness, and biosafety. An intelligent DDS composed of NIPAM and PLL in a composite molecular chain architecture is rationally designed and constructed. On this basis, quantum chemical calculations combined with multiscale molecular simulations are employed to elucidate in depth its self-assembly behavior, drug encapsulation mechanism, and multi-stimuli-responsive characteristics. The conformational transitions and drug release kinetics driven by variations in temperature and pH are systematically dissected. By incorporating multiphysics coupling mechanisms and electronic structure evolution analysis, this work seeks to overcome critical mechanistic bottlenecks in RES delivery at the molecular level and to provide a theoretical foundation and design strategy for the development of novel intelligent drug delivery platforms applicable to structurally analogous natural polyphenolic compounds.

## Results and discussion

### Conformational flexibility engineering enables the design of multi-responsive intelligent drug delivery architectures

**Figure 1:**
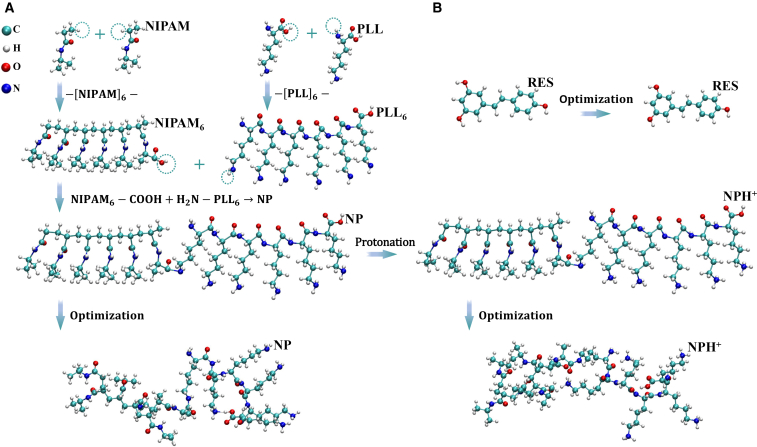
Structural modeling and quantum chemical optimization of NP, NPH^+^, and RES molecules (A) Construction of the composite molecular chain NP based on NIPAM and PLL units, followed by protonation of the PLL segment to generate the cationic form (NPH^+^). The panel illustrates the three-dimensional structural assembly of NP and NPH^+^, as well as their optimized conformations derived from quantum chemical calculations. (B) Initial three-dimensional configuration of the RES molecule and its energetically optimized stable conformation.

As illustrated in Figure 1A, NIPAM monomers (CH_2_ = CH-CONH-CH(CH_3_)_2_) undergo free radical polymerization, during which the C=C double bonds are cleaved and converted into C-C single bonds, resulting in a linear hexameric oligomer composed of six covalently linked units, [-CH_2_-CH(CONH-CH(CH_3_)_2_)-]_6_, herein denoted as NIPAM_6_. To enhance its subsequent conjugation reactivity, a terminal functionalization step is introduced by oxidizing a methyl group on the isopropyl side chain into a carboxyl group, yielding a carboxyl-terminated segment (NIPAM_6_-COOH). In parallel, PLL monomers (NH_2_-CH(COOH)-(CH_2_)_4_-NH_2_) are polymerized through condensation between the α-carboxyl and ε-amino groups to form linear hexapeptides via stable peptide bonds (-CO-NH-), with terminal hydrogens added to saturate the chain ends, denoted as PLL_6_. Subsequently, the NIPAM_6_-COOH segment is covalently coupled to the PLL_6_ chain through an amide linkage between the carboxyl and amino termini, yielding a composite molecular chain (NP) that integrates both thermoresponsive and pH-responsive functionalities. To simulate the weakly acidic microenvironments often present in pathological sites such as tumors or inflamed tissues,^34^ all α-amino groups in the PLL segment of NP are protonated (i.e., additional protons are introduced), producing a positively charged conformation referred to as NPH^+^. This modified structure is intended to represent the responsive behavior of the NP chain under physiologically relevant acidic conditions.

Based on the constructed molecular structures, full geometry optimization of both NP and NPH^+^ is performed using density functional theory (DFT). As shown in Figure 1A, the pre-optimization configurations exhibit relatively linear and orderly arrangements of the polymer segments. In contrast, the optimized structures display increased spatial expansion and pronounced asymmetry in both the backbone and side chains, with NPH^+^ in particular exhibiting marked chain bending and local folding. These conformational changes are primarily attributed to the enhanced electrostatic repulsion among positively charged groups introduced via protonation, which induces local tension and spatial rearrangement within the backbone. This structural evolution not only enhances the overall flexibility of the system but may also significantly influence its drug-loading capacity and stimuli-responsive release behavior. Notably, under pathological conditions with acidic microenvironments, the increased conformational plasticity and synergistic regulatory potential are expected to cf. superior responsiveness and dynamic control over therapeutic delivery.

In contrast, the optimized structure of the RES molecule (Figure 1B) reveals that its two phenolic rings remain highly coplanar before and after optimization, indicating excellent conformational stability. This rigid, planar architecture facilitates the formation of noncovalent interactions such as hydrogen bonding and van der Waals forces-during the subsequent encapsulation process, serving as a critical foundation for stable binding with nanocarriers. Overall, the conformational flexibility and tunable steric hindrance of the NP and NPH^+^ systems provide a critical structural foundation for their multivalent interactions with RES and enable the possibility of controllable drug encapsulation. The thermos-responsive conformational transitions of the NIPAM segments, combined with the charge-responsive properties of the PLL domains, are expected to facilitate precise modulation of drug release behavior under multifactorial physiological stimuli. These optimized structures serve as essential initial inputs for subsequent multiscale molecular simulations and thermodynamic/kinetic analyses, enabling in-depth exploration of their dynamic response mechanisms and drug delivery performance under diverse stimulus conditions.

### Complete RES encapsulation and conformational locking enabled by an NP-based nanocarrier system

To gain molecular-level insight into the spontaneous recognition and encapsulation behavior of the polyphenolic drug RES by NP molecules in aqueous solution, we construct a composite system comprising 60 NP molecules and 6 RES molecules (System-298 K-100 ns). All-atom molecular dynamics simulations are performed in a cubic water box under periodic boundary conditions at 298 K for 100 ns, aiming to explore the full progression of the nanocarrier system from initial dispersion to conformational stabilization under physiological temperature. Prior to the production run, the temporal evolution of key thermodynamic parameters—including potential energy, temperature, pressure, and density—during energy minimization and the NVT and NPT equilibration stages is systematically monitored. The results indicate that all critical thermodynamic parameters reach stable plateaus within reasonable fluctuation ranges, and no systematic drift is observed (see Figure S1 in the Supporting Information), demonstrating that the system undergoes sufficient relaxation and attains thermodynamic stability before entering the production phase.

**Figure 2:**
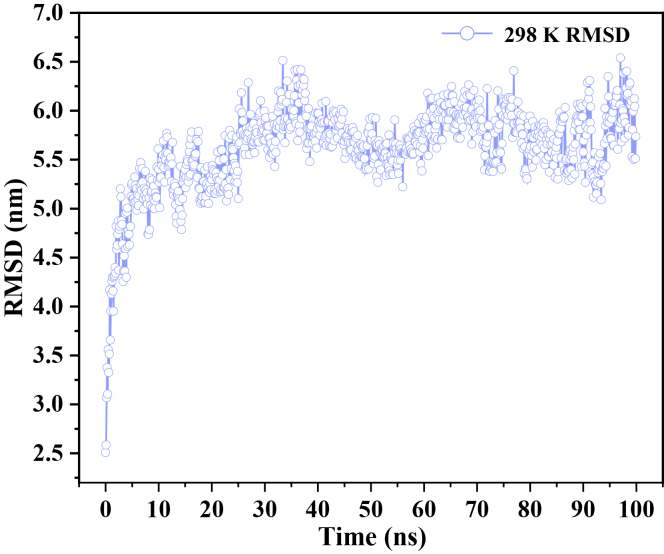
Root-mean-square deviation (RMSD) of the non-solvent molecular clusters in the studied system Time evolution of the RMSD for RES-NP clusters in the system-298 K-100 ns, reflecting the structural dynamics of the drug-loaded assembly.

On this basis, the trajectories obtained from the production stage are subjected to kinetic analysis. As shown in Figure 2, the root-mean-square deviation (RMSD) profile clearly reveals the self-assembly kinetics of the system: during the initial 0–35 ns, the RMSD increases sharply, indicating pronounced conformational rearrangement driven by noncovalent interactions between NP and RES, which promote spatial reorganization and aggregation. In the subsequent 35–100 ns interval, the RMSD progressively stabilizes, with minimal fluctuations particularly evident between 70 and 100 ns, suggesting that the system reaches a configurational steady state. This trend indicates that, following rapid cluster reconstruction at the early stage, the system evolves into a relatively stable conformational ensemble, thereby providing a reliable structural basis for subsequent analyses of drug-loaded configurations and molecular aggregation behavior.

**Figure 3:**
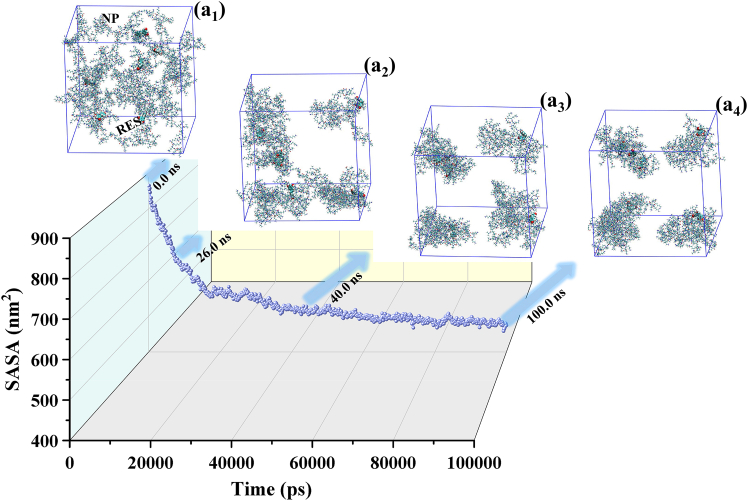
Solvent accessible surface area (SASA) and conformational evolution of non-solvent molecular clusters in the studied system Time-dependent changes in the SASA of the RES-NP clusters in the system-298 K-100 ns simulation, illustrating the dynamic features of molecular aggregation. (a_1_): initial configuration of the RES-NP cluster at 0.0 ns, with representative conformations at 26.0 ns, 40.0 ns, and 100.0 ns shown in (a_2_), (a_3_), and (a_4_), respectively. RES molecules are represented as colored spheres, and NP chains are displayed as colored sticks. For clarity, solvent molecules are omitted from the visualizations.

The variation in solvent-accessible surface area (SASA) serves as a key indicator of the molecular aggregation behavior within the system. As shown in Figure 3A, the SASA of the RES-NP complex decreases sharply during the initial phase of the simulation and stabilizes over the final 30 ns. This variation reflects the spontaneous aggregation of NP molecules driven by the hydrophobic segments on their surfaces in an aqueous environment, resulting in the formation of compact structures that effectively shield the interior of the system from direct contact with water and thereby significantly reduce the solvent-exposed surface area. Notably, RES continuously participates in this process, suggesting that it very likely becomes progressively embedded or encapsulated within the aggregation core through hydrophobic interactions, hydrogen bonding, or electrostatic adsorption. Such a spatially exclusive and stabilized architecture provides a robust structural foundation for achieving controlled drug release in subsequent stages.

Panels 3(a_1_)-(a_4_) further visualize the temporal evolution of system conformations throughout the simulation. At the initial time point (0.0 ns), RES and NP molecules are randomly dispersed in the aqueous phase, forming a loose and unaggregated configuration. By 26.0 ns, multiple NP-driven primary clusters emerge, with several RES molecules adsorbed onto their surfaces or embedded within, indicating a characteristic trend of cooperative aggregation. At 40.0 ns, cluster rearrangement continues, accompanied by positional migration of some RES molecules, leading to a more compact overall structure. By 100.0 ns, although local conformational adjustments persist, the number and spatial arrangement of clusters remain largely stable, consistent with the plateau observed in the RMSD and SASA curves during the later stage. Notably, in the final equilibrated state, all six RES molecules are effectively adsorbed or encapsulated within NP-based aggregates, demonstrating the nanocarrier’s high drug-loading completeness and molecular inclusion capacity, as well as its excellent conformational stability as a DDS.

The spatial distribution pattern of NP molecules around RES is further quantified using the radial distribution function (RDF),^35^ defined as:

(Equation 1) $$g_{RES-NP}\left(r\right)=\frac{\left\langle \rho_{NP}\left(r\right)\right\rangle }{{\left\langle \rho_{NP}\right\rangle }_{local}}\frac{1}{{\left\langle \rho_{NP}\right\rangle }_{local}}\frac{1}{N_{RES}}\int_{i\in RES}^{N_{RES}}\int_{j\in NP}^{N_{NP}}\frac{\delta \left(r_{ij}-r\right)}{4\pi r^{2}}$$

**Figure 4:**
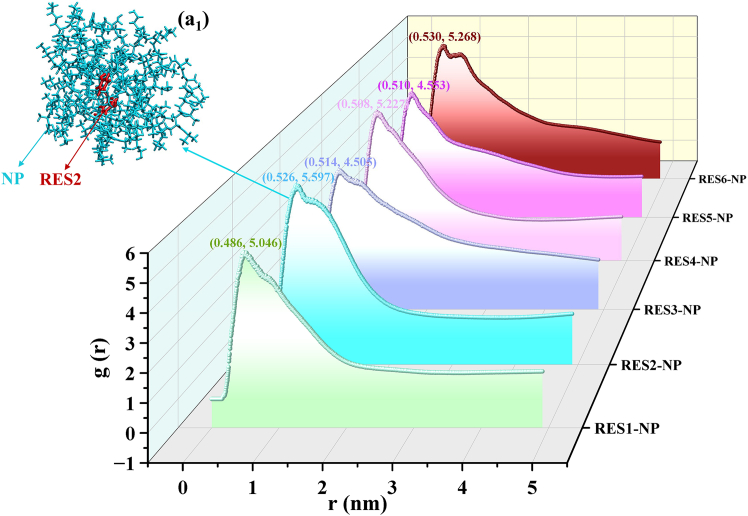
Spatial distribution of NP molecules relative to RES in the studied system Radial distribution function (RDF) curves of six RES molecules (labeled as RES1, RES2, RES3 … RES6) calculated with each RES molecule as the reference center, depicting the distance-dependent spatial correlations between individual RES molecules and surrounding NP molecules in the system-298 K-100 ns. The peak positions and intensities of each curve are annotated to facilitate quantitative comparison. (a_1_): Local structural snapshot, centered on RES2, extracted from the system-298 K-100 ns. The RES molecule is shown as dark brown sticks, and NP molecules are depicted in light green. This image, together with the RES2-NP RDF curve, provides a physical representation of the spatial distribution of NP molecules around RES2.

Here, ⟨*ρ*_*NP*_(*r*)⟩ denotes the local number density of NP components at a distance *r* from RES, and ${\left\langle \rho_{NP}\right\rangle }_{local}$ represents the average local density of NP components within a spherical shell of radius *r* centered on RES. Indices *i* and *j* indicate atomic indices, with the integration performed over all atoms. Essentially, *g*_*RES*-*NP*_(*r*) describes the probability of finding NP atoms within a shell of thickness *dr* at a distance *r* from the reference position of RES. For the system-298 K-100 ns, all six RES molecules (labeled RES1 through RES6) are individually selected as reference centers, and the RDFs of surrounding NP atoms are calculated accordingly. The resulting distributions are shown in Figure 4A.

As shown in Figure 4A, all RDF curves exhibit a pronounced peak at approximately 0.5 nm, indicating a high local concentration of NP molecules around the RES molecules at this distance, which corresponds to the so-called “first coordination shell” in a physical sense. This characteristic distance reflects the spatial scale at which effective interactions occur between RES and NP molecules. Figure 4(a_1_) presents a representative local conformation centered on RES2, where NP molecules form a densely packed shell surrounding it. Notably, the RDF peak corresponding to RES2 reaches the highest value (5.597), suggesting the strongest adsorption capacity for NP molecules. In contrast, RES3 shows the lowest peak value (4.505), indicating relatively weaker adsorption, though still maintaining a substantial level of encapsulation. All RDF curves exhibit similar peak positions and overall profiles, suggesting consistent structural environments across the six RES molecules and reflecting high uniformity in drug encapsulation performance, with no significant binding bias among individual RES molecules.

Overall, molecular dynamics simulations clearly demonstrate that NP and RES molecules undergo spontaneous, rapid, and stable aggregation in aqueous solution. The system completes molecular recognition and conformational reorganization within a short timescale, followed by the maintenance of a stable cluster architecture. The convergence of RMSD, RDF, and SASA results collectively confirms the thermodynamic stability of the drug-loaded state. Importantly, the system achieves 100% effective adsorption or encapsulation of RES, and its outstanding drug-loading capacity provides a solid foundation for further in-depth investigations into the controlled release mechanisms of RES.

### Deciphering the driving mechanisms and multiscale interaction networks governing the RES-NP system from initial recognition to stable encapsulation

**Figure 5:**
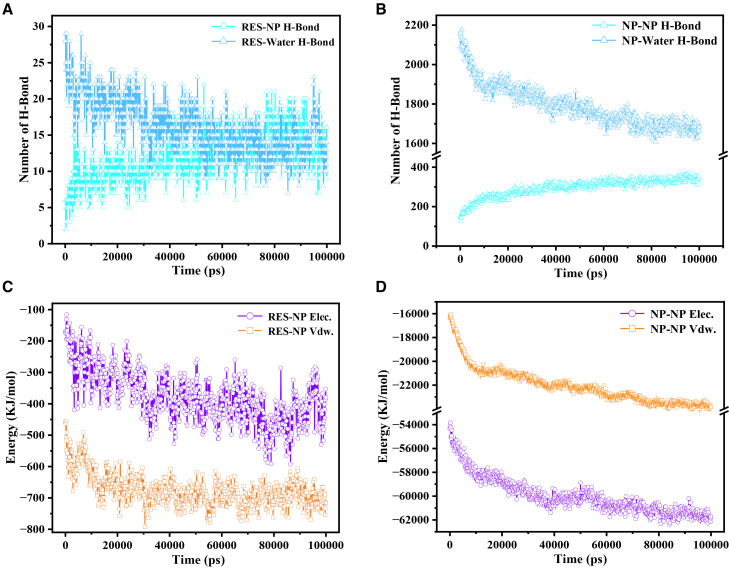
Temporal evolution of key intermolecular interactions in the system-298 K-100 ns (A) Time-dependent number of hydrogen bonds formed between RES and NP molecules (RES-NP), and between RES and water molecules (RES-water). (B) Number of hydrogen bonds between NP molecules (NP-NP) and between NP and water molecules (NP-water) over the course of the simulation. (C) Time profiles of electrostatic interaction energy (Elec.) and van der Waals interaction energy (Vdw.) between RES and NP molecules, and between RES and water molecules. (D) Temporal changes in electrostatic and van der Waals interaction energies between NP molecules.

To elucidate the microscopic driving forces underlying the encapsulation of RES by NP molecules, we perform a time-resolved analysis of the key noncovalent interactions within the system-298 K-100 ns. As shown in Figure 5A, the number of hydrogen bonds between RES and NP (RES-NP), as well as between RES and water (RES-Water), evolves markedly over the course of the simulation. A clear inverse trend is observed: while RES-NP hydrogen bonds steadily increase, RES-water hydrogen bonds decline significantly. This shift indicates that RES molecules gradually migrate from their initially dispersed, hydrated state toward the NP-enriched regions, where they become increasingly nested within NP clusters. This encapsulation process markedly enhances the frequency of molecular contacts between RES and NP, thereby promoting hydrogen bond formation. Concurrently, as RES molecules are progressively shielded from the aqueous environment, their interactions with surrounding water diminish. Meanwhile, as the NP molecules self-assemble into dense clusters, their proximity and mutual orientation become more favorable for intra-cluster hydrogen bonding, while their exposure to water decreases. As a result, the number of NP-NP hydrogen bonds increases significantly, whereas NP-Water hydrogen bonds are correspondingly reduced, as illustrated in Figure 5B.

Figure 5C shows the time-dependent evolution of electrostatic (Elec.) and van der Waals interaction energies (Vdw.) between RES and NP molecules. Both interaction energies remain negative throughout the simulation and exhibit a pronounced decline during the initial ∼35 ns, indicating a gradual transition of the system from a dispersed to a bound state. Throughout this process, electrostatic and van der Waals forces work in concert to guide the progressive association of RES and NP molecules, culminating in a nested architecture enabled by conformational matching and spatial reorganization. During the simulation, RES and NP molecules continuously adjust their positions and orientations within the simulation box as time progresses, aiming to drive the system toward a relatively low-energy configuration (transiently stabilized state) within an extremely short time window. This frequent conformational rearrangement is reflected in Figure 5C as pronounced fluctuations in the interaction energy curves over a short timescale.

Figure 5D illustrates the temporal evolution of interaction energies between NP molecules. Both Elec. and Vdw. remain at relatively large negative values throughout the simulation, indicating strong intermolecular attraction among NP molecules. This pronounced attractive interaction serves as the fundamental driving force for cluster formation. The continuous decrease in the NP-NP interaction energy reflects the self-assembly process of the cluster structure. Notably, all examined parameters, including hydrogen bond count, Elec., and Vdw., exhibit significant dynamic changes within the first ∼35 ns, after which they gradually stabilize. This critical inflection point marks the completion of the conformational transition from the initial dispersed state to a stable aggregated state, clearly delineating the kinetic timescale of the self-assembly process in the RES-NP system.

**Figure 6:**
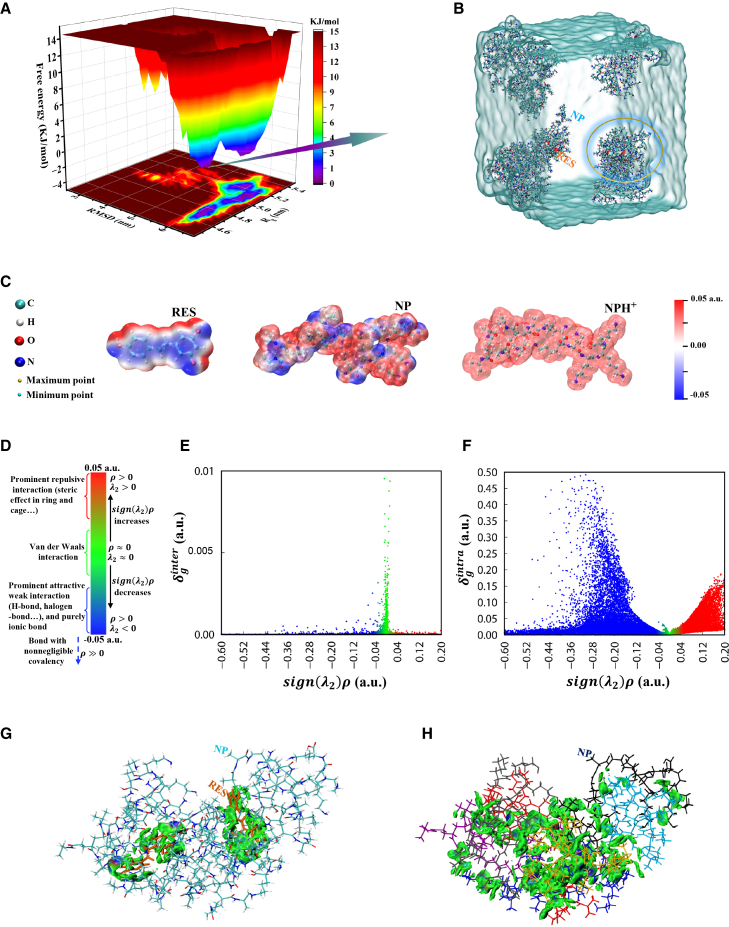
Multiscale mapping of essential intermolecular interactions in the system-298 K-100 ns (A) Three-dimensional Gibbs free energy landscape of the system-298 K-100 ns. (B) Representative molecular conformation corresponding to the lowest-energy basin in the free energy landscape. The water box is shown in light cyan, NP chains are depicted as colored sticks, and RES molecules are shown as colored spheres. (C) Molecular electrostatic potential surfaces of RES, NP, and NPH^+^ molecules, with blue, white, and red indicating regions of negative, neutral, and positive potential, respectively. Local maxima and minima are marked with golden and cyan spheres. (D) Correlation between different values of *sign*(*λ*_2_)*ρ* and corresponding interaction types, color-coded to distinguish interaction types and intensities. (E) The scatterplot of *δ*_*g*_($\delta_{g}^{inter}$) and *sign*(*λ*_2_)*ρ* values between the NP molecular network and RES molecules is generated, with points color-coded according to panel (d) to intuitively illustrate the types of interactions. (F) Scatterplot of *δ*_*g*_($\delta_{g}^{intra}$) versus *sign*(*λ*_2_)*ρ* values for both intermolecular and intramolecular regions within all molecules of the R-cluster. (G) Isosurface map of $\delta_{g}^{inter}$ in the contact regions between the NP molecular framework and RES molecules within the R-cluster. To facilitate visualization, RES molecules are shown in orange, and the values of *sign*(*λ*_2_)*ρ* are projected onto the isosurface using the color scale defined in (D), clearly highlighting the spatial distribution and types of interactions between the two molecular species. (H) Colored isosurface map of $\delta_{g}^{inter}$ between NP molecules within the R-cluster. Each NP chain is displayed in a distinct color to clearly distinguish the intermolecular interaction regions and types.

To gain detailed insights into the interaction regions, types, and strengths between molecules-and to elucidate the physicochemical basis of RES’s stable binding within the NP system-a multiscale interaction landscape of RES-NP recognition, adsorption, and encapsulation is constructed (Figure 6). Figure 6A presents the three-dimensional Gibbs free energy surface of the system-298 K-100 ns, revealing three distinct energy minima that correspond to several low-free-energy conformational states evolved during the simulation. The global minimum conformation, shown in Figure 6B, reveals that all RES molecules are effectively adsorbed or encapsulated by NP molecules, forming a highly ordered nanocluster architecture. This structural feature not only reconfirms the superior drug-loading capability of the NP system for RES, but also provides a representative structural basis for subsequent mechanistic analyses. Among all identified conformations, the cluster highlighted by the orange circle in Figure 6B is particularly representative, where two RES molecules are surrounded and nested within the cluster core by multiple NP chains, demonstrating enhanced stability through cooperative multivalent interactions. This local assembly (R-cluster) is therefore selected for further analysis to investigate the driving interactions and assess the intelligent release behaviors of the system under various stimuli.

During the processes of molecular recognition and initial adsorption, the distribution of electrostatic potential plays a critical role in mediating intermolecular interactions.^36^ According to the principle of electrostatic complementarity, positively charged regions tend to attract negatively charged areas, thereby forming initial adsorption sites.^37^ Figure 6C presents the electrostatic potential maps of RES, NP, and NPH^+^ molecules, generated by coloring the electron density isosurfaces. In RES, the front-facing regions of the two phenolic rings-comprising C-C, C=C, and C=O bonds-appear as highly negative zones (blue), whereas the peripheral hydrogen atoms at the molecular edges exhibit localized positive potential (red). Within NP molecular chains, nearly all oxygen and nitrogen atoms exhibit negative electrostatic regions, while the hydrogen atoms along the polymer backbone consistently show distinct positive potentials. Therefore, the interaction sites between RES and NP can be qualitatively classified into two categories: (1) the front-facing negatively charged regions of the RES phenolic rings interacting with the positively polarized hydrogen atoms located at the edges of NP chains, and (2) the side hydrogen atoms of RES associating with the locally negative O or N atoms in NP. Evidently, the former mode provides a relatively larger contact interface and thus exhibits a higher probability of occurrence, making it the predominant mode of initial molecular association. In a similar manner, the distribution of positively and negatively charged regions within the NP molecules themselves underpins their mutual electrostatic interactions and aggregation behavior, which are critical for the formation of a stable drug-loading system. As the simulation progresses, these electrostatically driven initial configurations continue to evolve through dynamic rearrangement of molecular positions, minimizing the total energy of the cluster and ultimately leading to the thermodynamically stable structure shown in Figure 6B.

In addition, two key descriptors are introduced to elucidate intermolecular interactions: the *sign*(*λ*_2_)*ρ* index from the atoms in molecules theory (AIM)^38^ and the δg function from the independent gradient model (IGM).^39^ Specifically, *sign*(*λ*_2_)*ρ* represents the product of the electron density (*ρ*) and the sign of the second largest eigenvalue (*λ*_2_) of the Hessian matrix of the electron density, which allows the qualitative differentiation of interaction types (see Figure 6D). The δg descriptor, on the other hand, quantifies the difference in electron density gradients and effectively identifies the interaction strength between atoms in adjacent fragments.^40^ The R-cluster structure shown in Figure 6B is divided into three fragments for further analysis: the NP molecular assembly as one fragment, and each of the two RES molecules as separate fragments. Interfragment interactions are characterized by computing *sign*(*λ*_2_)*ρ* and $\delta_{g}^{inter}$, and the results are projected onto a scatterplot (Figure 6E) using the color scale defined in Figure 6D. The plot reveals two prominent peaks: one composed of blue points centered around *sign*(*λ*_2_)*ρ* ≈ −0.04, and the other composed of green points near *sign*(*λ*_2_)*ρ* ≈ 0.00, corresponding to hydrogen bonding and van der Waals interactions, respectively. Notably, the broader area and higher intensity of the green peak indicate that van der Waals interactions serve as the dominant interfragment binding force between NP and RES molecules. Furthermore, intrafragment interactions ($\delta_{g}^{intra}$) are analyzed (Figure 6F). The green region near *sign*(*λ*_2_)*ρ* ≈ 0 corresponds to widespread intramolecular van der Waals interactions. The red points in the positive region indicates steric repulsion between conformational units, whereas the extensive blue region with significantly negative *sign*(*λ*_2_)*ρ* values corresponds to covalent bond regions, confirming the structural stability within each molecule.

To further visualize interfragment interactions, isosurface maps of $\delta_{g}^{inter}$ are generated with the corresponding *sign*(*λ*_2_)*ρ* values projected onto the surfaces using the color scale defined in Figure 6D. As shown in Figure 6G, the predominance of green regions, with local blue areas at the molecular contact interfaces, further confirms that the RES-NP interactions are mainly governed by van der Waals forces, supplemented by hydrogen bonding. In detail, these interaction regions primarily extend along the planes of the two phenolic rings of RES and their connecting backbone, with substantial contributions from the planar aromatic moieties of RES and the H atoms of various functional groups on NP. This observation supports the aforementioned hypothesis of electrostatic complementarity. Additionally, by treating each NP molecule within the R-cluster as an independent fragment, $\delta_{g}^{inter}$ isosurface maps and *sign*(*λ*_2_)*ρ* projections are generated to explore inter-NP interactions, as shown in Figure 6H. The results indicate that NP-NP attractions are also dominated by van der Waals forces. The presence of multiple localized O/N-H interaction sites further highlights the critical role of hydrogen bonding in stabilizing the cluster architecture. Collectively, these findings demonstrate that the cooperative network of weak interactions ensures the structural integrity of the drug-loaded assembly. More importantly, they offer a rational design basis for the future development of high-performance nanocarriers with tunable release behaviors by modulating solvent conditions, charge distributions, or drug molecular structures.

### Mechanistic elucidation of drug release driven by temperature-induced carrier cohesion and structural reconfiguration

**Figure 7:**
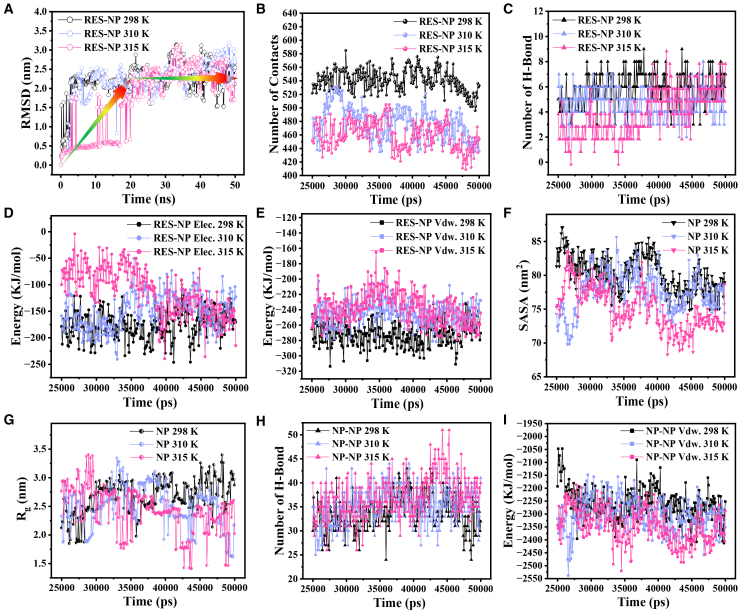
Dynamic evolution analysis of the R-cluster system over a 50 ns simulation at 298 K (room temperature), 310 K (physiological temperature), and 315 K (tumor/inflammatory site temperature) To facilitate comparison, simulation data at 298 K are shown in black, while those at 310 K and 315 K are displayed in light blue and light red, respectively. Except for RMSD, all other plots present time distributions during the equilibrium phase (final 25–50 ns of the simulation). (A) Time evolution of RMSD for the RES-NP molecular cluster. (B) Number of contacts between RES molecules and the NP cluster. (C) Number of hydrogen bonds between RES molecules and the NP cluster. (D) Elec. between RES molecules and the NP cluster. (E) Vdw. between RES molecules and the NP cluster. (F) SASA of the NP system. (G) Radius of gyration of the NP system. (H) Number of hydrogen bonds among NP molecules. (I) Vdw. among NP molecules.

To investigate the conformational stability and interaction evolution mechanisms of the R-cluster under different physiological and pathological temperatures, it is embedded in a cubic water box and subjected to all-atom molecular dynamics simulations for 50 ns at 298 K (ambient temperature), 310 K (normal body temperature), and 315 K (a characteristic elevation observed in tumor or inflamed tissues^41^). The corresponding systems are denoted as R-cluster-298 K-50 ns, R-cluster-310 K-50 ns, and R-cluster-315 K-50 ns, respectively. Figure 7A presents the RMSD time profiles of the RES-NP complexes at each temperature. All systems exhibit pronounced conformational rearrangement within the initial 25 ns, followed by a relatively stable fluctuation phase, indicating that the system reaches a configurational steady state within the simulated timescale. Accordingly, the data collected over the 25–50 ns interval are employed for subsequent analyses of RES-NP interactions and cluster stability, serving as the steady-state reference window.

To quantify the degree of proximity between drug molecules and nanocarriers within a specific distance range at each time point, a contact number function *N*_*C*_*(t)* is defined to calculate the number of atom pairs where the distance *r*_*i*_ between atom *i* in drug molecules and atom *j* in the nanoparticle is within 0.6 nm^42^:

(Equation 2) $$N_{C}\left(t\right)=\sum_{i=1}^{N_{RES}}\sum_{j=1}^{N_{NP}}\int_{r_{i}}^{r_{i}+0.6\;nm}\delta \left(r\left(t\right)-r_{j}\left(t\right)\right)dr$$

Here, *N*_*RES*_ and *N*_*NP*_ denote the total number of atoms in the RES and NP systems, respectively. Figure 7B shows the temporal evolution of the contact number at different temperatures. Compared to the condition at 298 K, the RES-NP contact number significantly decreases at 310 K and 315 K, suggesting that elevated temperatures attenuate the molecular association between the drug and the carrier. A similar trend is observed in the hydrogen bond count (Figure 7C), supporting the notion that temperature rise perturbs the hydrogen-bonding network between RES and NP.

Further analysis of the intermolecular electrostatic interaction energy (Elec.) and van der Waals interaction energy (Vdw.) (Figures 7D and 7E) reveals that both energy terms remain negative across all temperature conditions, indicating that attractive potentials dominate the system interactions. More critically, the absolute values of both Elec. and Vdw. continuously decrease with increasing temperature, suggesting an expansion of the RES-NP intermolecular distance and a concomitant weakening of interaction strength. These results collectively form a highly consistent body of evidence, demonstrating that elevated temperature markedly diminishes the adhesive forces between RES and NP.

Panels (F)–(I) of Figure 7 reveal the temperature-responsive characteristics of the NP system. As shown in Figure 7F, the SASA value decreases with increasing temperature, indicating a reduction in the overall volume of the NP cluster. A similar trend is observed for the radius of gyration (Rg) in Figure 7G, suggesting that the NP conformation becomes more compact upon heating. Concurrently, the number of hydrogen bonds among NP molecules increases significantly at elevated temperatures (Figure 7H), and the absolute value of Vdw. rises accordingly (Figure 7I), reflecting enhanced inter-molecular attraction and intensified aggregation within the system. This temperature-responsive behavior is primarily attributed to the aggregation-state transition of the NIPAM segments in the NP structure. At lower temperatures, the NIPAM chains adopt a relatively loose conformation due to weak interactions between the hydrophilic amide groups and the hydrophobic isopropyl groups. Once the temperature exceeds the low critical solution temperature (LCST), hydrophobic interactions drive the isopropyl groups to associate, triggering a phase transition and the formation of a compact hydrophobic core.^43^^,^^44^

**Figure 8:**
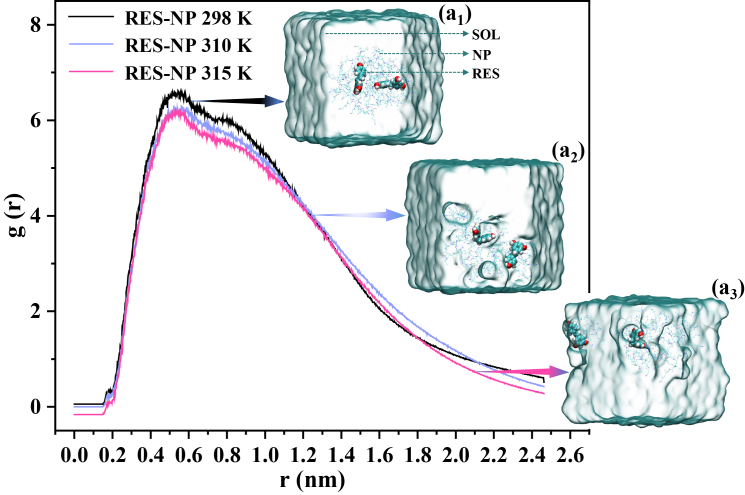
Temperature-dependent spatial distribution of NP relative to RES in the R-cluster system Radial distribution functions of NP molecules with respect to RES at 298 K, 310 K, and 315 K, with RES designated as the reference center. Curves corresponding to different temperatures are distinguished by color to facilitate comparative observation. (a_1_)–(a_3_): Representative equilibrium conformations of the simulated systems at 298 K, 310 K, and 315 K, respectively, which correspond to the distribution curves shown in (a) and collectively describe the relative spatial arrangement between NP and RES molecules.

The structural transformation of the NP cluster alters the spatial distribution of RES molecules within the system. As shown in Figure 8A, the radial distribution functions of NP molecules with respect to RES are presented at different temperatures. All three curves exhibit similar peak profiles; however, with increasing temperature, the intensity of the main peak gradually decreases, and its position slightly shifts to the right. This is because the overall condensation of NP clusters causes the RES molecules to gradually deviate from the center of the NP clusters and even be ejected, thereby weakening the distribution density of NP molecules around RES (reflected by the weakened peak intensity) and increasing the characteristic distance between RES and NP (manifested as a rightward peak shift). These findings are further corroborated by representative equilibrium conformations shown in Figure 8 (a_1_)–(a_3_): at 298 K, RES molecules are embedded within the NP cluster, with NP molecules evenly distributed around them; at 310 K, RES molecules are displaced toward the periphery of the cluster; and at 315 K, one RES molecule adheres to the cluster surface, while the other is almost completely dissociated from the aggregate.

In summary, the multidimensional data collectively demonstrate that, under physiologically relevant temperature elevation, the NP system undergoes enhanced aggregation, volume contraction, and increased compactness. These structural transitions alter the spatial arrangement of interaction sites between RES and NP molecules, leading to weakened RES-NP adhesion and ultimately resulting in drug dissociation. It is important to emphasize that the selected temperature range (298–315 K) does not aim to represent macroscopic differences in overall body temperature between tumor and normal tissues. Rather, it is designed to characterize the molecular-scale response of thermoresponsive polymer-based delivery systems to subtle thermal perturbations in the vicinity of physiological temperature, particularly near the LCST region. For stimulus-responsive materials possessing phase-transition thresholds, temperature variations on the order of only a few K are significantly amplified at the molecular level, thereby triggering conformational compaction, interaction reorganization, and functional state transitions.^45^ On this basis, the present study elucidates, at the molecular scale, a temperature-driven intelligent release mechanism governed by carrier structural rearrangement, thereby providing a physicochemical foundation for the rational design of thermoresponsive nanodrug delivery systems.

### Protonation-induced structural expansion and interaction decoupling reveal the pH-responsive drug release pathway

**Figure 9:**
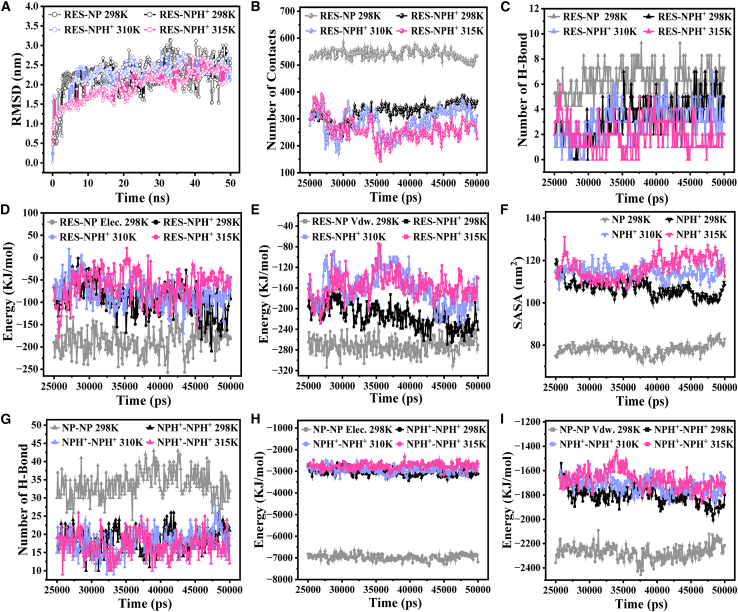
Dynamic evolution analysis of the R-cluster system after protonation at 298 K, 310 K, and 315 K over a 50 ns simulation period Protonation is performed on all NP molecular chains within the R-cluster system, resulting in positively charged conformations denoted as NPH^+^. To facilitate the assessment of protonation effects, data from the non-protonated R-cluster-298 K-50 ns system are included as gray curves for reference. Except for RMSD, all other data represent time distributions within the equilibrium phase (25–50 ns). (A) Time-dependent RMSD profile of RES-NPH^+^ cluster at different temperatures. (B) Number of contacts between RES molecules and the NPH^+^ cluster. (C) Number of hydrogen bonds between RES molecules and the NPH^+^ cluster. (D) Elec. between RES molecules and the NPH^+^ cluster. (E) Vdw. between RES molecules and the NPH^+^ cluster. (F) SASA of the NPH^+^ system. (G) Number of hydrogen bonds among NPH^+^ molecules. (H) Elec. among NPH^+^ molecules. (I) Vdw. among NPH^+^ molecules.

To simulate the influence of the acidic microenvironment typical of tumor or inflamed tissues on drug release behavior, a comprehensive protonation treatment is applied to all NP molecules in the R-cluster system, yielding positively charged conformations (NPH^+^), which are subsequently solvated in a constructed cubic water box. Molecular dynamics simulations are then performed under conditions of 298 K, 310 K, and 315 K for 50 ns, and the resulting systems are denoted as R-clusterH^+^-298 K-50 ns, R-clusterH^+^-310 K-50 ns, and R-clusterH^+^-315 K-50 ns, respectively. Figure 9 presents the characteristic parameters describing the structural evolution of these systems. To facilitate comparison and reveal the driving effect of protonation on structural dynamics, the behavior of the non-protonated reference system R-cluster-298 K-50 ns is also included in Figure 9 as a baseline, represented by gray curves. As shown in Figure 9A, the RMSD profiles of both protonated and non-protonated systems exhibit highly consistent trends, with a rapid increase during the first 25 ns followed by a plateau, indicating that the systems reach relatively stable conformations after 25 ns. Accordingly, all subsequent analyses in Figures 9B–9I are based on data obtained from the equilibrium period (25–50 ns).

The contact behavior between the drug molecules and the carrier cluster is quantitatively characterized in Figure 9B. In all R-clusterH^+^ systems, the number of contacts between RES and NPH^+^ is markedly reduced compared to that in the non-protonated R-cluster system, suggesting that protonation substantially weakens the binding capacity between the drug and the carrier. This change indicates the occurrence of potential drug release events. Figure 9C further confirms this trend, showing that the number of hydrogen bonds between RES and NPH^+^ is significantly reduced relative to that in the RES-NP system. In parallel, both the Elec. (Figure 9D) and Vdw. (Figure 9E) between RES and NPH^+^ exhibit markedly lower absolute values compared to those in the non-protonated system, indicating a widespread attenuation of intermolecular affinity. In addition to protonation, the synergistic effect of elevated temperature is also clearly captured. As shown in Figures 9B–9E, increasing the temperature from 298 K to 315 K leads to further decreases in the number of contacts, hydrogen bonds, and interaction energies between RES and NPH^+^, indicating that higher temperature facilitates the dissociation of drug-carrier interactions. However, the extent of these changes is considerably smaller than that induced by protonation, highlighting the dominant role of pH stimulus in regulating drug loading behavior.

Figure 9F reveals that the SASA of the NPH^+^ system is significantly larger than that of the NP system, indicating a substantial volumetric expansion and structural relaxation of the protonated molecular cluster. Furthermore, temperature elevation from 298 K to 315 K enhances this expansion trend, albeit to a lesser extent than protonation, suggesting a synergistic effect between thermal excitation and protonation. Figures 9G–9I systematically demonstrate the fundamental impact of protonation on the aggregation behavior of the carrier molecules. Compared with the non-protonated NP system, the NPH^+^ system exhibits markedly reduced numbers of intermolecular hydrogen bonds, as well as diminished absolute values of Elec. and Vdw., reflecting a general weakening of intermolecular cohesion. This is consistent with the increased SASA, which implies expanded intermolecular spacing and supports a protonation-induced decohesion mechanism. Moreover, the temperature-dependent curves of the NPH^+^ system show that elevated temperatures further attenuate hydrogen bonding, Elec., and Vdw. to varying degrees, reinforcing the notion that thermal effects facilitate the loosening of the molecular packing within the carrier system.

The aggregation behavior changes induced by protonation can be traced back to alterations at the electronic structure level. A retrospective examination of the molecular electrostatic potential map in Figure 6C, the introduction of additional protons at the amino termini of the five PLL chains in NPH^+^ generates a uniformly distributed positive charge, rendering the molecular surface almost continuously covered with weak positive potential. This electronic reconfiguration substantially weakens the original electrostatic attractions between chains, and even introduces slight electrostatic repulsion. As a result, the side chains tend to stretch and the molecular conformations become more relaxed, both intramolecularly and intermolecularly, leading to a marked reduction in aggregation capacity. This phenomenon accounts for the macroscopic volumetric expansion observed in the NPH^+^ system. In addition, the temperature-driven aggregation observed in the NP system is mediated by interactions between locally positive and negative electrostatic potential regions along the NIPAM chains. By contrast, the nearly homogeneous positive charge distribution in NPH^+^ molecules disrupts this mechanism. Consequently, the NPH^+^ system not only fails to exhibit the thermally induced compaction observed in NP, but instead undergoes progressive de-aggregation upon heating, consistent with the physical principle of thermal expansion and contraction.^46^ The structural expansion of NPH^+^ directly affects its drug-loading capability. The increased intermolecular distances and weakened cohesion hinder the ability of NPH^+^ chains to maintain effective encapsulation of RES molecules, thereby rendering drug diffusion into the surrounding environment possible.

**Figure 10:**
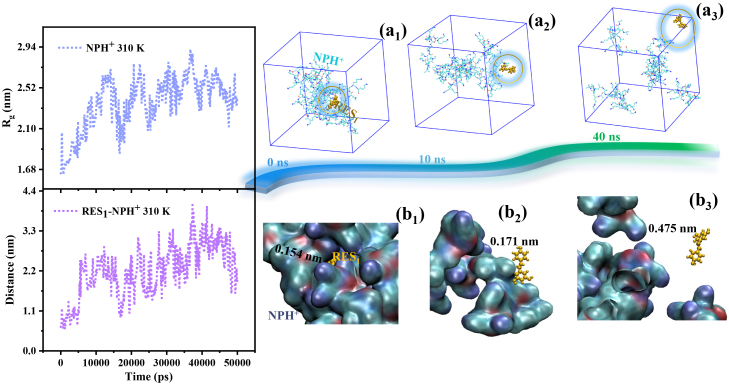
Structural variation analysis of the R-clusterH^+^-310K-50 ns system Temporal evolution of the radius of gyration of the NPH^+^ molecular cluster (top) and the time-dependent distance between the drug molecule labeled as RES_1_ and the NPH^+^ cluster (bottom). (a_1_)–(a_3_): Representative conformations of the system at 0 ns, 10 ns, and 40 ns, respectively. Solvent molecules are omitted for clarity. The drug molecule RES_1_ is highlighted in orange using a stick-and-ball representation to facilitate the observation of its spatial relationship with the cluster. (b_1_): Magnified view of the local structure surrounding RES_1_ and NPH^+^, corresponding to the orange-circled region in (a_1_). The NPH^+^ molecular system is shown using the QuickSurf representation, and the minimum distance between RES_1_ and NPH^+^ is indicated. (b_2_) and (b_3_) similarly display the enlarged views of the highlighted regions in (a_2_) and (a_3_), respectively.

Figure 10 presents representative snapshots of structural evolution, using the R-clusterH^+^-310 K-50 ns system as an example. In Figure 10A, the radius of gyration of the NPH^+^ molecular cluster (light blue curve) initially increases over time and subsequently fluctuates around a stable level, indicating a gradual loss of compactness during the early stage of the simulation and a transition toward a more relaxed morphology. The conformations captured at representative time points in Figure 10(a_1_)–(a_3_) further corroborate this conclusion. These snapshots reveal that the NPH^+^ molecules progressively shift from an initially compact and tightly associated state to a more dispersed configuration, with certain molecular chains exhibiting detached and uncorrelated distributions.

The purple curve in Figure 10A illustrates the time-dependent distance between the center of mass of the dynamically monitored drug molecule labeled as RES_1_ and that of the NPH^+^ cluster. A marked increase in this distance is observed during the first half of the trajectory, followed by a fluctuating plateau. The representative conformations shown in Figure 10(a_1_)–(a_3_) visually capture this dynamic process, with RES_1_ highlighted in orange to facilitate the observation of its positional relationship with the NPH^+^ cluster. Additionally, the orange-circled local regions in Figure 10(a_1_)–(a_3_) are magnified in the corresponding panels Figure 10(b_1_)–(b_3_), where the minimum atomic distances between RES_1_ and NPH^+^ are explicitly labeled. Notably, at the onset of the simulation, RES_1_ is almost completely encapsulated within the NPH^+^ cluster. Over time, its position gradually shifts toward the periphery and eventually approaches a nearly dissociated state. Collectively, Figure 10 constructs a dynamic, molecular-level “drug release landscape,” underscoring the direct correlation between protonation-induced loosening of the carrier structure and the de-encapsulation of the drug. This provides direct evidence for understanding the pH-responsive behavior of the DDS at the atomic scale.

Throughout this study, we systematically elucidate, at the molecular level, the recognition, encapsulation, and controllable release behaviors of RES within the thermo- and pH-responsive smart nanocarrier system, thereby constructing a comprehensive “intercalation-reconstruction-disassociation” drug delivery landscape. This work offers an effective strategy for enhancing the bioavailability of RES. The results demonstrate that the engineered NP molecular chains collaboratively stabilize RES encapsulation at ambient temperature through electrostatic, hydrogen bonding, and van der Waals interactions. Upon temperature elevation, the NIPAM segments undergo hydrophobic aggregation, triggering cluster rearrangement and drug disengagement. Under acidic conditions, protonation of the PLL chains induces strong electrostatic repulsion, which drives the disassembly of the nanocluster and facilitates efficient drug release. In addition, this study provides an in-depth analysis of key interaction sites, optimal stimulus conditions, molecular response windows, and conformational transition pathways by examining electrostatic potential distributions, free energy landscapes, and interatomic interaction metrics. These insights yield essential design parameters and structural optimization directions for the development of clinically oriented multi-responsive DDS platforms and establish a theoretical paradigm for the intelligent delivery of RES-analogous natural compounds in complex pathological environments.

### Limitations of the study

Several limitations of this study should be acknowledged. First, the molecular models and simulation protocols employ fixed polymer chain lengths and specific compositional ratios to construct the NIPAM-PLL nanocarriers and to investigate their encapsulation and release behavior toward RES. While these settings are sufficient to capture the essential conformational transitions and interaction mechanisms underlying dual thermo-pH responsiveness, variations in chain length, component ratio, or drug loading concentration may quantitatively influence cluster stability and release kinetics. A systematic exploration of this broader parameter space is beyond the scope of the present work. Second, the current investigation focuses primarily on temperature and pH as the dominant stimuli governing drug release. Other microenvironmental factors that may coexist in pathological tissues, such as ionic strength variations, redox conditions, or enzymatic activity, are not explicitly considered. In addition, the conclusions are mainly derived from multiscale molecular simulations and quantum chemical calculations. Experimental synthesis and validation of the proposed nanocarrier system have not yet been performed, which may limit the direct assessment of the predicted behaviors under *in vivo* or *in vitro* conditions. Despite these limitations, the present study provides a detailed molecular-level understanding of a stimulus-driven “recognition-reconstruction-release” mechanism, offering a solid theoretical foundation for subsequent parameter optimization, multi-stimulus integration, and experimental validation of intelligent delivery systems.

## Resource availability

### Lead contact

Further information and requests for resources and reagents should be directed to and will be fulfilled by the lead contact, Yunpeng Luan (luanteam@163.com).

### Materials availability

This study did not generate new unique reagents.

### Data and code availability

• All original data reported in this paper have been deposited at Mendeley data and are publicly available as of the date of publication. The DOI is listed in the key resources table.
• This paper does not report original code.
• Any additional information required to reanalyze the data reported in this paper is available from the lead contact upon request.

## Acknowledgments

This work was financially supported by the Basic Research Project of Yunnan Province (no. 202501AT070335), the TCM Joint Project of Yunnan Province (no. 202301AZ070001-033), the Open Research Found Program of Yunnan Key Laboratory for Dai and Yi Medicines (no. 2024JS2406), the 10.13039/501100001809National Natural Science Foundation of China (nos. 82460988 and 32160223), the Science and Technology Plan Project of Yunnan Provincial Department of Science and Technology (no. 202401AZ070001-120), and the Reserve Talent Programme for Young and Middle-aged Academic and Technical Leaders of Yunnan Province (no. 202105AC160047).

## Author contributions

Writing - original draft, visualization, validation, methodology, investigation, formal analysis, data curation, funding acquisition, conceptualization: Q.S.; methodology, formal analysis, data curation: Z.L.; investigation, software, methodology, formal analysis, data curation: W.Z.; software, methodology, formal analysis, data curation: X.H.; investigation, data curation, supervision: P.H.; writing - review & editing, validation, methodology, conceptualization: Y.Q.; writing - review & editing, validation, methodology, conceptualization: L.L.; writing - review & editing, supervision, project administration, methodology, funding acquisition, conceptualization: Y.L.

## Declaration of interests

The authors declare no competing interests.

## STAR★Methods

### Key resources table

**Table:** 

REAGENT or RESOURCE	SOURCE	IDENTIFIER
**Deposited data**		

The structure data of the simulation system	This paper; Mendeley Data	https://doi.org/10.17632/2n8y74czds.1
Data for analyzing self-assembly and encapsulation processes under ambient conditions and for simulating conformational evolution and drug release mechanisms under thermal and pH-responsive stimuli.	This paper; Mendeley Data	https://doi.org/10.17632/2n8y74czds.1

**Software and algorithms**		

PubChem website	National Center for Biotechnology Information, National Institutes of Health, USA	https://pubchem.ncbi.nlm.nih.gov/docs/submissions
GaussView version 6.0	Gaussian Inc., USA	https://gaussian.com/gaussview6/
ORCA version 6.0	Max Planck Institute for Coal Research, Germany	https://orcaforum.kofo.mpg.de/app.php/portal
VMD version 1.9.3	Theoretical and Computational Biophysics Group, University of Illinois at Urbana-Champaign, USA	https://www.ks.uiuc.edu/Research/vmd/
Multiwfn version 3.8 (dev)	Beijing Kein Research Center for Natural Sciences, China	http://sobereva.com/multiwfn/
Sobtop version 1.0 (dev3.1)	Beijing Kein Research Center for Natural Sciences, China	http://sobereva.com/soft/Sobtop
GROMACS version 2022.1	GROMACS Development Team, Royal Institute of Technology and Uppsala University, Sweden	https://www.gromacs.org/
xTB version 6.6.1	Stefan Grimme Group, Mulliken Center for Theoretical Chemistry, University of Bonn, Germany	https://xtb-docs.readthedocs.io
GROMACS commands used to extract the simulation data	This paper; Mendeley Data	https://doi.org/10.17632/2n8y74czds.1

### Experimental model and study participant details

Our study does not use experimental models typical in the life sciences, it is computational simulation research.

### Method details

#### System construction and initial structure optimization

##### Molecular models and force field parameters

The initial three-dimensional structural coordinates of all molecules employed in this study are obtained from the PubChem database,^47^ including the NIPAM monomer, PLL monomer, and RES. A 6-mer oligomer of NIPAM (denoted as NIPAM_6_) and a 6-mer oligomer of PLL (PLL_6_) are constructed using Gaussian View.^48^ Subsequently, through amide bond coupling (carboxyl-amine covalent linkage), the terminal carboxyl group of NIPAM_6_ is covalently connected to the terminal amino group of PLL_6_, yielding a composite molecular chain (NIPAM_6_-PLL_6_, denoted as NP) that integrates thermoresponsive and pH-responsive potential within a unified framework. Considering that the pKa of the ε-amino groups on the PLL side chains is typically around 10, their protonation degree approaches thermodynamic completeness in the mildly acidic microenvironment associated with tumors or inflammation (pH ≈ 5.5–6.5). Accordingly, fully protonated PLL_6_ is adopted as a reasonable approximation to simulate pH-responsive behavior under acidic stimulation, and the corresponding molecular chain is denoted as NPH^+^. The detailed molecular construction procedure is illustrated in Figure 1. For force field parameterization, all molecular species (RES, NP, and NPH^+^) are assigned parameters generated using Sobtop software^49^ based on the GAFF force field framework.

##### Structure optimization and charge fitting

To obtain reliable initial structural parameters for molecular dynamics simulations, systematic quantum chemical calculations and charge fitting procedures are performed for RES, NP, and NPH^+^. Considering the broad applicability of the B3LYP functional^50^ combined with D3(BJ) dispersion correction^51^ and the def2-TZVP(-f) basis set^52^^,^^53^ in biomolecular modeling—and its favorable balance between computational efficiency and physical reliability—geometry optimizations are carried out at this level of theory using the ORCA package.^54^ The resulting stable conformations are presented in Figure 1. Based on the optimized geometries, the electrostatic potential (ESP) is calculated on the van der Waals surface. The obtained ESP data are subsequently fitted to RESP2(0.5) atomic charges using the Multiwfn program.^55^ These high-precision RESP2(0.5) charges replace the original charges in the standard GAFF force field, thereby constructing initial RES, NP, and NPH^+^ systems that more accurately reflect the underlying electronic distribution. Within this framework, the NP (or the NPH^+^) composite molecular chain is regarded as the minimal functional unit of the multi-responsive polymer system. Its local conformational variations and interaction patterns constitute the molecular foundation for the macroscopic architecture and stimulus-responsive behavior of the DDS.

#### Molecular dynamics simulation protocol

##### Simulation systems

To systematically investigate the loading and stimulus-responsive release behavior of RES, three categories of molecular dynamics simulation systems are constructed. First, the System-298K-100ns model is established. In this system, 60 NP molecules and 6 RES molecules are randomly distributed within a cubic simulation box of 10 × 10 × 10 nm^3^. The system is solvated using an explicit water model and simulated for 100 ns at 298 K to explore the spontaneous drug-loading process between RES and the carrier under ambient conditions. Second, a representative drug-loaded cluster is extracted from the final configuration of the above simulation. This cluster is re-solvated in a smaller cubic box (5 × 5 × 5 nm^3^), forming the Cluster series systems. To probe thermoresponsive characteristics, independent 50 ns simulations are performed at 298 K, 310 K, and 315 K, denoted as Cluster-298K-50ns, Cluster-310K-50ns, and Cluster-315K-50ns, respectively. Finally, to examine the dual thermal–pH response mechanism, all NP molecules within the drug-loaded cluster are protonated to generate NPH^+^, yielding the ClusterH^+^ series systems. These systems are simulated for 50 ns in a 5 × 5 × 5 nm^3^ box at 298 K, 310 K, and 315 K, and are correspondingly designated as ClusterH^+^-298K-50ns, ClusterH^+^-310K-50ns, and ClusterH^+^-315K-50ns. All aforementioned systems are solvated using a four-point water model (PIP4P) to ensure an accurate description of water-water and solute-water interactions.

##### Simulation parameters and procedures

All molecular dynamics simulations are performed using the GROMACS software package.^56^ Electrostatic interactions are treated with the particle mesh Ewald (PME) method, and a cutoff radius of 1.2 nm is applied for Lennard–Jones interactions.^56^ All covalent bonds involving hydrogen atoms are constrained using the LINCS algorithm, and an integration time step of 2 fs is employed throughout the simulations. Each system undergoes the following sequential protocol. First, energy minimization is conducted using the steepest descent algorithm until the maximum force in the system falls below 1000 kJ/mol/nm. Subsequently, NVT ensemble equilibration is performed for 1000 ps, during which the system is relaxed to the target temperature using the V-rescale thermostat.^57^ This is followed by 1000 ps of NPT ensemble equilibration, where the pressure is stabilized at 1.0 bar using the Berendsen barostat.^57^ Finally, the production phase is carried out in the NPT ensemble for the designated simulation time (100 ns or 50 ns). During this stage, temperature and pressure are maintained using the V-rescale thermostat and the Berendsen barostat, respectively.

#### Trajectory analysis and post-processing calculations

After confirming that the RMSD of each system reaches equilibrium, the terminal segments of the production trajectories are selected for systematic analysis (0–100 ns for System-298K-100ns and the final 25 ns for all other systems). All conventional structural, dynamical, and energetic analyses are performed using the standard tools implemented in GROMACS. Specifically, periodic boundary conditions are corrected using gmx trjconv; RMSD are calculated with gmx rms; the radius of gyration and solvent-accessible surface area are obtained using gmx gyrate and gmx sasa, respectively; radial distribution functions are evaluated with gmx rdf; hydrogen bonds are analyzed via gmx hbond; electrostatic and van der Waals interaction energies are extracted using gmx energy; and intermolecular contact numbers are determined through gmx mindist.

To further elucidate the intrinsic nature of intermolecular interactions from an electronic-structure perspective, the representative drug-loaded cluster extracted from System-298K-100ns is subjected to single-point energy calculations at the GFN2-xTB level.^58^ The resulting wavefunctions are analyzed using the independent gradient model method implemented in Multiwfn,^59^ generating colored δg (electron density gradient difference function) isosurfaces. Visualization is performed using VMD.^60^ In addition, the surface ESP distributions of RES, NP, and NPH^+^ are computed based on Multiwfn-derived data and rendered using VMD.

### Quantification and statistical analysis

Trajectory-derived quantities including RMSD, radius of gyration, solvent accessible surface area, radial distribution functions, hydrogen bond numbers, and interaction energies were calculated using standard analysis tools implemented in GROMACS. All values are reported as time-dependent distributions derived from simulation trajectories.

### Additional resources

Our study has not generated or contributed to a new website/forum, and it is not part of a clinical trial.
