# Supplementary material for: Thermo-pH dual-responsive nanocarriers enable multiscale-controlled resveratrol release via molecular remodeling and electronic decoupling
Source: iScience. 2026 Mar 23;29(4):115440. doi: 10.1016/j.isci.2026.115440 (PMC13087792; doi:10.1016/j.isci.2026.115440)
Supplement: Document S1. Figure S1 [file mmc1.pdf]

iScience, Volume *29*

Supplemental information

Thermo-pH dual-responsive nanocarriers enable multiscale-controlled resveratrol release via molecular remodeling and electronic decoupling

Qijiang Shu, Zedong Lin, Wenjuan Zhao, Xiaokun Hua, Pengru Huang, Yong Qiu, Li Li, and Yunpeng Luan

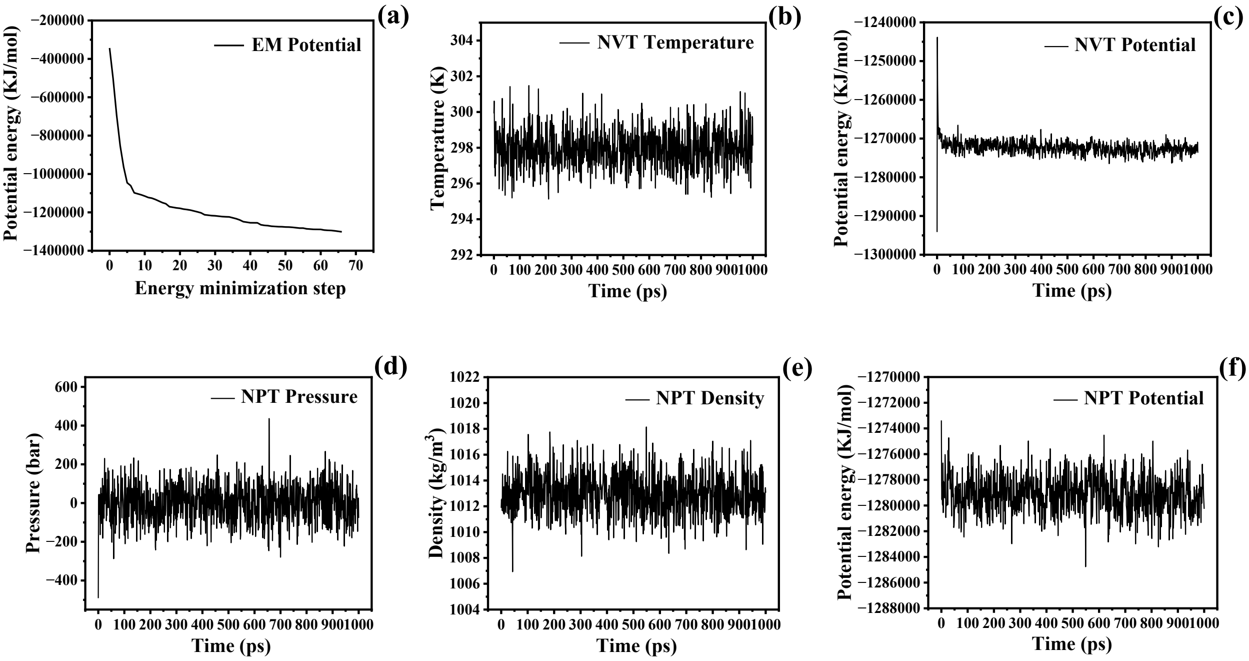

Figure S1. Time evolution of key thermodynamic parameters during the pre-equilibration stages of the System-298K-100ns prior to production molecular dynamics simulations, Related to Figure 2. (a) Potential energy profile during the energy minimization stage. The potential energy decreases rapidly and converges to a stable value, indicating that unfavorable contacts in the initial configuration are effectively eliminated and the system enters a low-energy conformational region; (b) Temperature as a function of time during the NVT equilibration stage. The temperature remains stably around the target value of 298 K, with fluctuations within the expected statistical range, indicating effective temperature coupling; (c) Potential energy evolution during the NVT equilibration stage. After a brief relaxation period, the potential energy fluctuates slightly around a stable plateau without observable systematic drift; (d) Pressure evolution during the NPT equilibration stage. The pressure fluctuates around 1 bar; although instantaneous fluctuations are present, no persistent deviation is observed, consistent with the statistical fluctuation characteristics of a finite system; (e) Density variation during the NPT equilibration stage. The density remains stable at approximately 1013 kg/m³, close to the experimental density of liquid water, indicating that the system volume and solvent environment are effectively equilibrated; (f) Potential energy evolution during the NPT

equilibration stage. The potential energy maintains a stable plateau without any discernible trend. Collectively, these results demonstrate that the system reaches structural and thermodynamic stability prior to the production molecular dynamics simulations, thereby providing reliable initial conditions for subsequent conformational evolution and interaction analyses.
